# Expanding the role of proteasome homeostasis in Parkinson’s disease: beyond protein breakdown

**DOI:** 10.1038/s41419-021-03441-0

**Published:** 2021-02-04

**Authors:** Mingxia Bi, Xixun Du, Qian Jiao, Xi Chen, Hong Jiang

**Affiliations:** Department of Physiology, Shandong Provincial Key Laboratory of Pathogenesis and Prevention of Neurological Disorders and State Key Disciplines: Physiology, School of Basic Medicine, Medical College, Qingdao University, Qingdao, China

**Keywords:** Neural ageing, Neuronal physiology

## Abstract

Proteasome is the principal hydrolytic machinery responsible for the great majority of protein degradation. The past three decades have testified prominent advances about proteasome involved in almost every aspect of biological processes. Nonetheless, inappropriate increase or decrease in proteasome function is regarded as a causative factor in several diseases. Proteasome abundance and proper assembly need to be precisely controlled. Indeed, various neurodegenerative diseases including Parkinson’s disease (PD) share a common pathological feature, intracellular protein accumulation such as α-synuclein. Proteasome activation may effectively remove aggregates and prevent the neurodegeneration in PD, which provides a potential application for disease-modifying treatment. In this review, we build on the valuable discoveries related to different types of proteolysis by distinct forms of proteasome, and how its regulatory and catalytic particles promote protein elimination. Additionally, we summarize the emerging ideas on the proteasome homeostasis regulation by targeting transcriptional, translational, and post-translational levels. Given the imbalanced proteostasis in PD, the strategies for intensifying proteasomal degradation are advocated as a promising approach for PD clinical intervention.

## Facts

- Proteasome homeostasis undergoes dynamic and reversible regulation at transcriptional, translational, and post-translational levels.
- The vicious cycle between proteasome dysfunction and protein accumulation plays a key role in PD pathogenesis.
- Regulation of proteasome homeostasis serves as a promising approach in PD therapies.
- The development of PROTAC opens up new potentials for PD treatment based on proteasome modulation.

## Open questions

- A number of possible hybrid proteasomes arise in cells. What are the distinct functions of singly or doubly capped proteasomes?
- What are the mechanisms by which different transcription factors regulate a separate set of proteasome subunits?
- Is there a unified mechanism of protein aggregates and neurodegeneration triggered by proteasome dysfunction in Parkinson’s disease?
- How prevalent and what is the significance of proteasome activation-based approach for PD therapies?

## Introduction

Proteasome is a ubiquitous and highly plastic multisubunits complex responsible for protein degradation in both cytosol and nucleus. Since its initial discovery in 1987^1,2^, proteasome has been under intensive investigation to explore the inherent logic of proteasome function, including the recognition of ubiquitylated substrates, deubiquitylation of potential targets, translocation of unfolded proteins into the catalytic chamber, and the peptidase features for selective proteolysis^3–5^. Although widely assumed to be a single entity, there also exists various types of proteasome in cells, such as 26 S proteasome (constitutive proteasome), immunoproteasome, thymoproteasome, and spermatoproteasome^6,7^. Under certain conditions, specific proteasome is induced to perform different biological functions, while the efforts to distinguish these proteasomes and analyze their respective roles have not been accomplished. Indeed, proteasome is not a stable and static complex serving as a “cellular trashcan”^8^. The expression of proteasome undergoes dynamic and reversible regulation not only at the transcriptional level but also at the translational and post-translational level, such as phosphorylation, ubiquitylation, ADP-ribosylation, O-linked N-acetylglucosamine (O-GlcNAc), acetylation, and S-glutathionylation. Even with this information, a comprehensive understanding of the regulatory mechanisms underlying proteasome homeostasis remains elusive.

Proteasome activity becomes gradually impaired during aging and various neurodegenerative disorders including Parkinson’s disease (PD). The compromise of proteasomal degradation leads to aberrant protein aggregates, such as α-synuclein, which in turn binds to proteasome correlated with a reduction in protease activity^9,10^. These observations indicate a vicious cycle between proteasome dysfunction and protein accumulation in PD pathogenesis. It is thus inferred that proteasome is expected to be an attractive target for PD intervention^11,12^. Enhancing proteasome function by either increasing subunits assembly or activating 20 S gate opening might be useful for the clearance of protein aggregates. Consistent with this evidence, several small-molecule compounds have been postulated to be an effective approach in PD treatment. The development of proteolysis-targeting chimera (PROTAC) represents a burgeoning field and a new therapeutic regimen owing to the capacity of inducing a specific protein degradation, as well as its small-molecule nature and targeting “undruggable” proteins^13,14^. Additionally, PROTAC provides the opportunity to rapidly degrade proteins of interest, which effectively prevents the compensatory recovery due to the deletion of target proteins^13,15^. Even so, PROTAC is still an emerging technology, and its future development is also fraught with challenges.

This article reviews recent advances in the multiple regulation of proteasome homeostasis that ensures efficient protein degradation. Major new insights into proteasome activation have also emerged, which not only seems important in regulating protein turnover, but also provides potential application in drug discovery to combat the proteotoxicity in PD.

## Components of proteasome

Proteasome, as a multisubunits complex, accounts for the vast majority (at least 80%) of protein degradation. The best known 26 S proteasome, also named standard or constitutive proteasome, is composed of a 20 S core particle (CP or 20 S complex) attached to one or both ends by a 19 S regulatory particle (RP, 19 S complex or PA700). The 19 S complex, as a proteasome activator, is incorporated into two parts: the lid and the base. The lid consists of nine subunits, that is, Rpn3, Rpn5, Rpn6, Rpn7, Rpn8, Rpn9, Rpn11, Rpn12, and Rpn15. The base contains six ATPase subunits (Rpt1-6), as well as Rpn1, Rpn2, Rpn10, and Rpn13 (Table 1). The alternative proteasome activators, including 11 S regulator complex PA28 and PA200 (PSME4), substitute 19 S RP to assemble various forms of proteasome. Three members of PA28 have been identified, namely PA28α (PSME1), PA28β (PSME2), and PA28γ (PSME3). In contrast to PA28α and PA28β which form PA28αβ hetero-heptamer distributed throughout the cytoplasm, PA28γ forms a homo-heptamer predominantly located in the nucleus. Many studies have implicated PA28αβ in the production of major histocompatibility complex class I (MHC I) antigen peptides, and PA28γ has been known to conduct proteasomal degradation of numerous intact proteins^16,17^.

**Table 1:** Proteasome subunits names and function.

Subcomplex	*Saccharomyces cerevisiae*	*Homo sapiens*	Function
19 S lid	Rpn3	PSMD3	Structural
	Rpn5	PSMD12	Structural
	Rpn6	PSMD11	Structural
	Rpn7	PSMD6	Structural
	Rpn8	PSMD7	Structural
	Rpn9	PSMD13	Structural
	Rpn11	PSMD14	Deubiquitinase, Ub removal
	Rpn12	PSMD8	Structural
	Rpn15	PSMD9	Structural
19 S base	Rpn1	PSMD2	Ubp6 and Ub/Ubl binding
	Rpn2	PSMD1	Structural
	Rpn10	PSMD4	Initial Ub/Ubl binding
	Rpn13	ADRM1	
	Rpt1	PSMC2	ATPase subunits, substrates binding, unfolding, and translocation
	Rpt2	PSMC1	
	Rpt3	PSMC4	
	Rpt4	PSMC6	
	Rpt5	PSMC3	
	Rpt6	PSMC5	
20 S α-subunits	α1	PSMA6	20 S gate opening
	α2	PSMA2	
	α3	PSMA4	
	α4	PSMA7	
	α5	PSMA5	
	α6	PSMA1	
	α7	PSMA3	
20 S β-subunits	β1	PSMB6	Caspase-like activity
	β1i	PSMB9	Chymotrypsin-like activity
	β2	PSMB7	Trypsin-like activity
	β2i	PSMB10	Trypsin-like activity
	β3	PSMB3	
	β4	PSMB2	
	β5	PSMB5	Chymotrypsin-like activity
	β5i	PSMB8	Chymotrypsin-like activity
	β6	PSMB1	
	β7	PSMB4	
Associated DUBs	Ubp6	USP14	Deubiquitinase, 19 S activation
	—	UCH37	

**Fig. 1:**
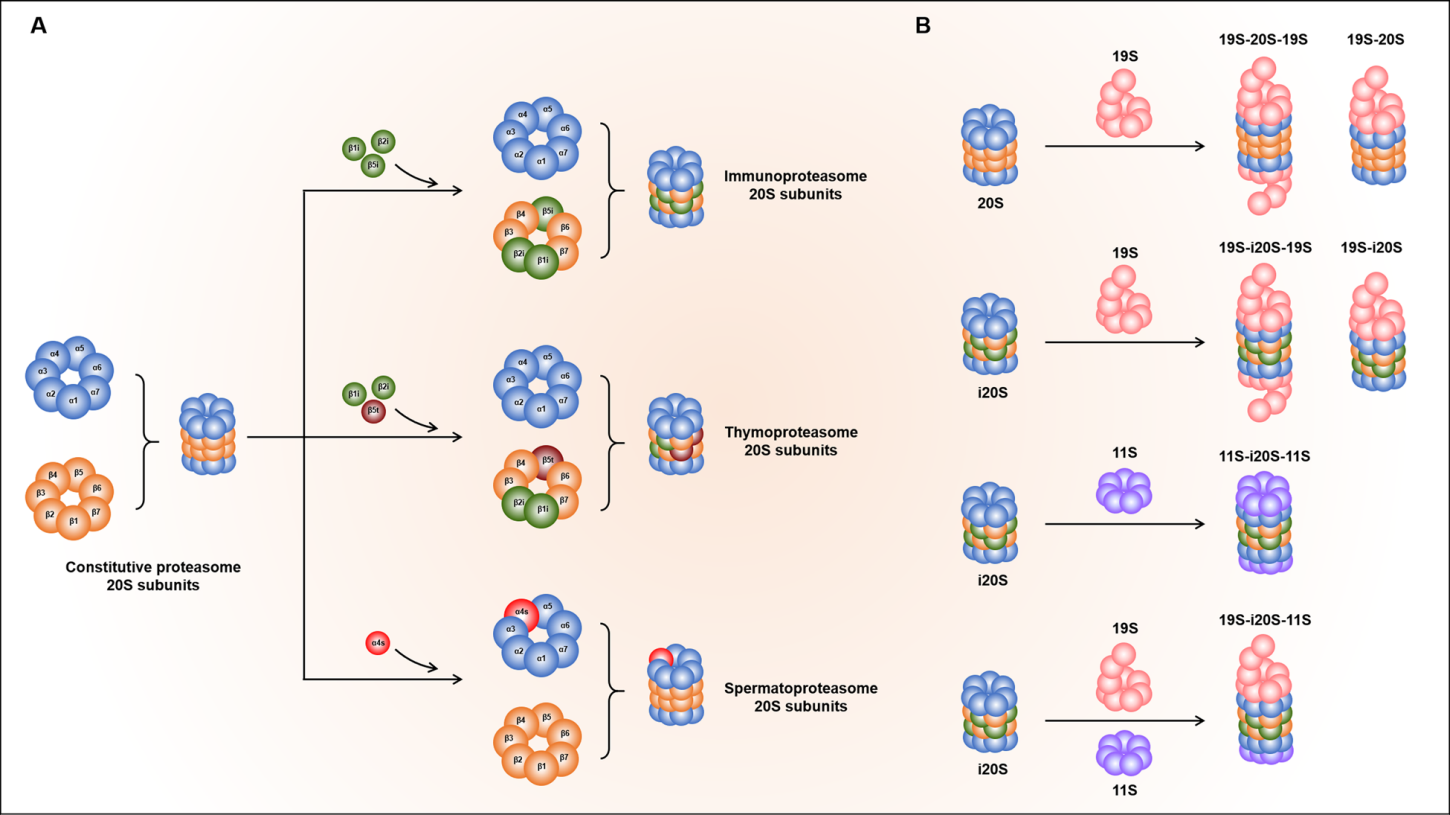
Different types of proteasome. **A** Three catalytic subunits β1, β2, and β5 subunits can be replaced by their homologues β1i, β2i, and β5i to form immunoproteasome, β5t substitutes β5 to form thymoproteasome, and α4s subunits replace their constitutive counterparts α4 to form spermatoproteasome. **B** The combination of 20 S CP with proteasome activator. The 19 S or 11 S complex can associate at one or both ends of 20 S to form different types of proteasome.

The 20 S complex is a barrel-shaped hollow cylindrical complex with 28 subunits arranged into four heptameric rings, the two outer rings composed of α_1–7_ subunits and the two inner rings composed of β_1–7_ subunits. Of note, the proteolytic activities are carried out by three β subunits with peptidase sites, namely β1, β2, and β5, which possesses the caspase-like, trypsin-like, and chymotrypsin-like activities, respectively. Upon certain stimulus, three catalytic subunits β1, β2, and β5 subunits can be replaced by their homologues β1i, β2i, and β5i to form immunoproteasome^18^, β5t substitutes β5 to form thymoproteasome^19^, and α4s subunits replace their constitutive counterparts α4 to form spermatoproteasome^20^. Currently, a number of possible hybrid proteasomes arise in cells because of the attachment of different proteasome activators to one or both sides of 20 S CP (Fig. 1). Nonetheless, the specific function of singly or doubly capped proteasomes remains to be elucidated.

## Regulation of proteasome homeostasis

### Transcriptional regulation of constitutive proteasome

**Fig. 2:**
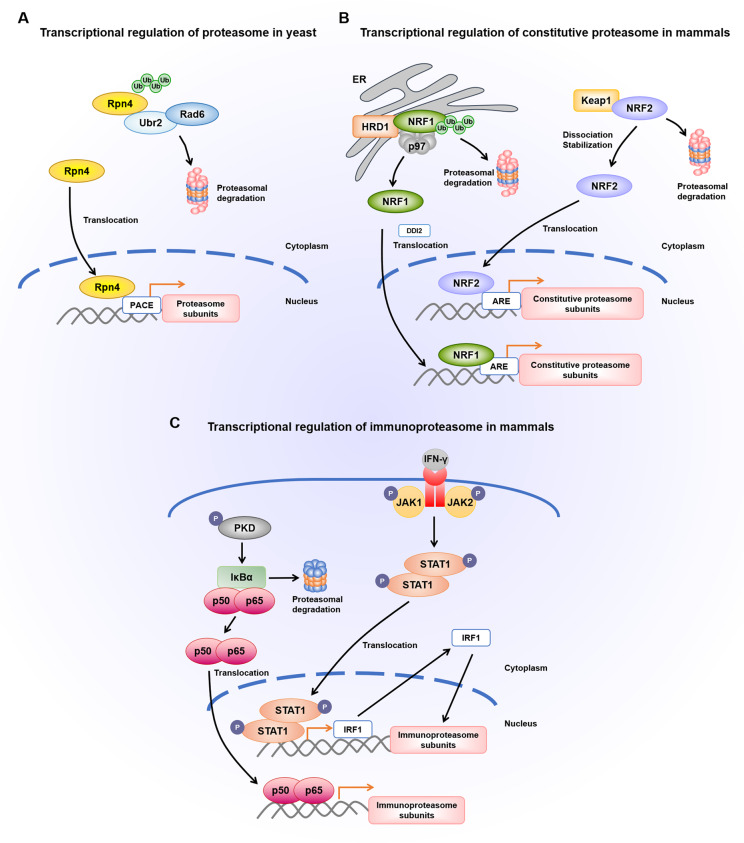
Transcriptional regulation of proteasome expression. **A** Transcriptional regulation of proteasome in yeast. Rpn4 serves as a transcription factor with short half-life (t1/2 ~2 min) owing to the proteasomal degradation mediated by E2 ubiquitin conjugating enzyme Rad6 and E3 ubiquitin ligase Ubr2. Upon proteasome inhibition, Rpn4 is translocated into the nucleus, where it binds to PACE sequence in the promoters of proteasome subunit genes, resulting in the compensatory increase of proteasome expression. **B** Transcriptional regulation of constitutive proteasome in mammals. NRF1 resides in the ER, which is degraded via ERAD pathway requiring ER-resident ubiquitin ligase HRD1 and ATPase p97. When the proteasome is inhibited, NRF1 is cleaved by DDI2, and then translocated to the nucleus where it binds ARE and activates the transcription of proteasome genes. During oxidative stress, NRF2-Keap1 complex disassociates, and NRF2 translocates into the nucleus to transcriptionally regulate proteasome expression. **C** Transcriptional regulation of immunoproteasome in mammals. Under the stimulation of IFN-γ, activated JAK1 and JAK2 leads to the dimerization and phosphorylation of STAT1, which translocates into the nucleus and binds to IRF1. Following translation, IRF1 moves back into the nucleus to increase the transcription of immunoproteasome. Upon oxidative injury, phosphorylated protein kinase D (PKD) leads to the disassociation of IκBα from NF-κB. In turn, IκBα is degraded and NF-κB translocates into the nucleus to regulate the expression of immunoproteasome.

In 1999, Mannhaupt et al. discovered a unique activating sequence (5’-GGTGGCAAA-3’) called proteasome-associated control element (PACE), which is localized in the promoters of most proteasome subunits^21^. In yeast, Rpn4 acts as a common transcription factor binding to PACE of genes encoding proteasome subunits to maintain the normal abundance of proteasome^21–23^. Notably, Rpn4 is extremely short-lived (t_1/2_ ~2 min) because the N-terminal region of Rpn4 contains a portable degradation signal for the continual proteasomal degradation. As a consequence, Rpn4 augments the synthesis of proteasome subunits in response to proteasome inhibition, which yields a negative feedback circuit. By contrast, Rpn4 deletion in yeast disrupts the expression of proteasome, therefore cells become more susceptible to various stimulus such as DNA damage and oxidative stress^24^. In yeast, transcription factor Rpn4 is indispensable for the compensatory increase of proteasome subunits to cope with stress conditions (Fig. 2).

In mammals, there also exists the transcriptional regulation of proteasome expression once proteasome function is impaired (Fig. 2). Despite the homologues of Rpn4 in mammalian cells have not been found, several transcription factors are implicated to fulfill the function of Rpn4. The expression of proteasome genes, including six CP subunits (α2, α5, α7, β3, β4, and β6) and five RP subunits (Rpt1, Rpt5, Rpt6, Rpn10, and Rpn11), is tightly regulated by nuclear transcription factor Y (NF-Y). Knockdown of NF-Y remarkably downregulates proteasome genes expression and inhibits cellular proteasome function^25^. Forkhead box O4 (FOXO4), an insulin/insulin-like growth factor-I (IGF-I) responsive transcription factor, amplifies proteasome activity by modulating Rpn6 expression in human embryonic stem cells^26^. Signal transducer and activator of transcription 3 (STAT3), which is activated through Janus kinase (JAK)/STAT pathway, transcriptionally regulates the expression of β5^27^. In addition, nuclear factor erythroid 2-related factor 2 (NRF2) is initially implicated to induce β5 expression upon exposure to oxidative stress^28^. Later, antioxidant response elements (AREs) sequences have been found in the 5’-untranslated region of 20 S proteasome subunits genes^29^. Intriguingly, a later study ascribes the induction of proteasome biogenesis to NRF1^30^. Echoing these results, brain-specific *Nrf1*^-/-^ knockout mice exhibit the downregulation of proteasomal genes, accompanied by the accumulation of polyubiquitylated proteins and age-dependent neurodegeneration^31^.

### Transcriptional regulation of immunoproteasome

Unlike the constitutive proteasome, immunoproteasome subunits lack functional ARE binding sequences. The *LMP2* gene encoding β1i subunit contains a bidirectional promoter characterized by the lack of TATA box and the presence of several GC boxes, which are likely the transcriptional start sites. Multiple transcription factors including signal transducer and activator of transcription 1 (STAT1)/interferon regulatory factor 1 (IRF1) dimers, nuclear factor κB (NF-κB), SP-1, AP-1, cAMP responsive element binding protein (CREB), and Zif268 (also known as Egr1), are involved in the *LMP2* (β1i) gene expression^32^. Furthermore, the promoter regions of *LMP7* and *MECL-1* encoding β5i and β2i, respectively, also contain NF-κB consensus sequence, cAMP regulatory elements, along with SP-1 and IRF1 binding sites^18,33,34^. In this case, the transcriptional regulation of β1i, β2i, and β5i share the relatively similar mechanisms. Upon interferon-γ (IFN-γ) stimulation, the activation of JAK1 and JAK2 causes the dimerization and phosphorylation of STAT1, which translocate into the nucleus and combine with IRF1 to promote its transcription. Then, IRF1 migrates back into the nucleus to stimulate the expression of immunoproteasome subunits. In addition, a potential alternative manner for immunoproteasome regulation is through NF-κB pathway. Upon oxidative injury, the phosphorylation of protein kinase D (PKD) disassociates IκBα from NF-κB. Upon the degradation of IκBα by the proteasome, NF-κB can translocate into the nucleus triggering the transcription of immunoproteasome subunits (Fig. 2).

### Translational regulation of proteasome

The activation of yeast mitogen-activated protein kinase (MAPK) Mpk1 followed by target of rapamycin complex 1 (TORC1) inhibition facilitates a rapid rise in the expression of RP assembly chaperones (RACs) and proteasome subunits. This process of proteasome homeostasis regulation is evolutionarily conserved in mammals. ERK5 (also known as MAPK7), the mammalian orthologue of Mpk1, also mediates the upregulation of RACs and proteasome abundance upon mammalian target of rapamycin complex 1 (mTORC1) inhibition^35^. Considering that neither the mRNA levels nor the protein stability of proteasome subunits are altered in response to the inhibition of TORC1/mTORC1, the regulation of proteasome by Mpk1/ERK5 probably occurs at the translational level^36^. It is clear that mTORC1 serves as a master regulator of proteasome abundance, whereas the relationship between mTORC1 and proteasome homeostasis seems to be controversial. Another study reveals that mTORC1 activation promotes the efficiency of proteasome-mediated protein degradation by increasing cellular proteasome content^37^. In this regard, it will be necessary to resolve the discrepancy of how mTORC1 affects the proteasome homeostasis under the particular cellular conditions.

## Post-translational modifications of proteasome

### Phosphorylation

In 1989, Haass and Kloetzel first reported the possibility that the phosphorylation of proteasome subunits had an impact on proteolytic activities during the *Drosophila* development^38^. In the following years, phosphorylation proteomics have shown a great deal of phosphorylation sites, which exist in almost every proteasome subunit^8^. Protein kinase A (PKA) was probably the first reported kinase involved in the phosphorylation of proteasome subunits^39^. Subsequent studies have shown that PKA directly phosphorylate Rpt6 at Ser120 and Rpn6 at Ser14, leading to the increased proteasomal peptidase activities^40–42^. PKA activation enhances the capacity of proteasome and promotes the elimination of protein aggregates^41^. In rat spinal cord neurons, PKA-mediated increased proteolytic activities reduce the accumulation of ubiquitylated proteins and protect cells from inflammatory injury^43^. Ca^2+^/calmodulin-dependent protein kinase II (CAMKIIα) directly phosphorylates Rpt6 at Ser120 and stimulates proteasome activity^44,45^. Mutation of Rpt6 at Ser120 blocks proteasome-dependent regulation of synaptic plasticity in the hippocampus^46,47^ (Table 2). Pharmacological inhibition of CaMKIIα abolishes the increase in proteolytic activity and the initiation of memory reconsolidation process^48,49^.

**Table 2:** An overview of proteasome related post-translational modifications.

Modifications	Targets	Enzymes	Functions	References
Phosphorylation	Rpn1 (S361)	UBLCP1	Proteasome activity ↑; 26 S proteasome assembly ↑	^146^
	Rpn2 (Y273)	p38 MAPK	Proteasome activity ↓; ubiquitylated protein degradation ↓	^52^
	Rpn6 (S14)	PKA	Proteasome activity ↑; ubiquitylated protein degradation ↑	^41,42^
	Rpt3 (T25)	DYRK2	Proteasome activity ↑; substrate translocation and degradation ↑; cell proliferation and tumorigenesis	^50,51^
	Rpt6 (S120)	PKA, CAMKIIα	Proteasome activity ↑; synaptic plasticity, learning and memory	^40,41^
	α4 (Y153)	c-Abl/Arg	Proteasome activity ↓	^53^
	α4 (Y106)	c-Abl/Arg	α4 degradation ↓; proteasome abundance ↑	^54^
	α7 (S243, S250)	CK2	Stabilizing RP-CP interaction; Ecm29 binding ↑	^55,147^
Ubiquitylation	Rpn10	Ube3c, Rsp5, Ubp2	Proteasome activity ↓; ubiquitylated conjugates affinity ↓	^56,57^
	Rpn13	Ube3c	Ubiquitylated protein degradation ↓	^58^
	α2	ALAD	Substrates entry into 20 S CP ↓	^59^
ADP-ribosylation	Nuclear proteasome	PARP	Proteasome activity ↑; oxidatively histone degradation ↑; cytokine-mediated neuroinflammation ↓	^60,61^
O-GlcNAc	Rpt2	OGT, OGA	Proteasome activity ↓; ubiquitylated protein degradation ↓	^65^
Acetylation	α6, β3, β6, β7	HDAC	Proteasome activity ↑	^67^
S-glutathionylation	Rpn1, Rpn2	—	Proteasome activity ↓	^148^
	α5, α6, α7	Glutaredoxin 2, thioredoxins 1/2	20 S gate opening ↑; oxidized and unfold protein degradation ↑	^149,150^

Dual-specificity tyrosine-regulated kinase 2 (DYRK2) phosphorylates a particular site of the proteasome, Rpt3 Thr25, leading to the enhanced substrate translocation and degradation^50,51^. Under osmotic stress conditions, p38 MAPK negatively regulates the proteasome activity by phosphorylating Rpn2 at Thr273, so as to induce the accumulation of polyubiquitinated proteins^52^. The nonreceptor tyrosine kinases c-Abl and Arg directly phosphorylate α4 at two tyrosine residues, Tyr106 and Tyr153. α4 Tyr153 phosphorylation inhibits proteasome activity, whereas Tyr106 phosphorylation suppresses the degradation of α4, therefore upregulating proteasome abundance^53,54^. These findings suggests the dual roles of c-Abl/Arg in the proteasome homeostasis and activity. Moreover, casein kinase 2 (CK2) phosphorylates α7 at Ser243 and Ser250, which appears to stabilize the RP-CP interaction and promote the binding of proteasome quality control factor Ecm29^55^ (Table 2).

### Ubiquitylation

Rpn10 is the first proteasome subunit identified as a proteasome substrate, as well as a major target of ubiquitylation. It has been shown that Rpn10 undergoes the proteasomal degradation facilitated by E3 ubiquitin ligase Ube3c^56^. Indeed, Ube3c endows proteasomes with the capacity to extend the ubiquitin chains on targets, which can be disassembled by a proteasome-associated deubiquitinating enzymes (DUB), Ubp6. Moreover, Isasa et al. have reported that Rpn10 is ubiquitylated by Rsp5 and deubiquitylated by Ubp2. Under stress conditions, Rpn10 monoubiquitylation is reduced, which in turn rescues proteasome function by enhancing the proteolytic activity, providing a stress sensitive mechanism to control proteasome catalytic activity^57^. Additionally, when proteasome function is inhibited by bortezomib, Rpn13 becomes extensively and selectively polyubiquitylated by E3 ligase Ube3c, which strongly inhibits the proteasome’s ability to degrade ubiquitin conjugates, but not hydrolyzing peptides and non-ubiquitylated proteins^58^ (Table 2). It has also been reported that delta-aminolevulinic acid dehydratase (ALAD) serves as an endogenous proteasome inhibitor, which is associated with the ubiquitylation of α2 subunit, thereby inhibiting the entry of substrates into 20 S catalytic chamber^59^. Although the consequence of proteasome subunits ubiquitylation under different conditions remain a subject for further study, this modification appears to be valuable to evaluate the proteasome function.

### ADP-ribosylation

ADP-ribosylation is the transfer of ADP-ribose moiety from NAD^+^ to target proteins. The functional interaction of poly(ADP-ribose) with 20 S proteasome leads to a specific enhancement of the peptidase activity. ADP-ribosylation activates nuclear 20 S proteasome to efficiently degrade oxidatively modified histone proteins, which is proposed as an adaptive response to oxidative defense^60^. Given the important role of ADP-ribosylation in DNA repair, the activation of proteasome is accompanied by the degradation of oxidized histones in the nucleus, which will otherwise make DNA repair impossible^61^. Besides, proteasome modification via ADP-ribosylation participates in cytokine-mediated neuroinflammation. Poly(ADP-ribose) polymerase (PARP) enables activated microglia to resist oxidative injury through an upregulation of the nuclear proteasome activity, resulting in the enhanced protein turnover and degradation of oxidatively modified proteins^62^. Of note, PARP inhibition impairs activated, but not resting microglia owing to the reduced proteasomal degradation, which might be beneficial, particularly in PD in which activated microglia acts as a pathogenic factor. Considering that glial cells are involved in the maintenance of neuronal function, PARP might be taken into consideration with the intention of ensuring neuronal survival.

### O-Glycosylation

O-GlcNAc is a regulatory post-translational modification in which O-GlcNAc transferase (OGT) adds GlcNAc monosaccharides to the hydroxyl groups on serine or threonine residues, while O-GlcNAcase (OGA) removes this modification^63^. Both OGT and OGA are abundant in the brain, with the highest expression in the hippocampal granular and pyramidal neurons and cerebellar Purkinje cells^64^. Pharmacological inhibition of OGA activity by streptozotocin or OGA knockdown leads to a rapid accumulation of O-GlcNAc and inhibition of proteasome function, thereby exacerbating neuronal apoptosis. Exposure of 26 S, but not 20 S, proteasome to OGT impairs the proteolysis of transcription factor SP-1 and a hydrophobic peptide owing to the inhibition of 19 S ATPase activities. The degradation of ubiquitinylated proteins requires the opening of 20 S CP probably by Rpt2, whose O-GlcNAc modification correlates inversely with the proteasome activity^65^ (Table 2). In addition, the biotin-cystamine tag strategy identifies six O-GlcNAc sites within the murine 20 S CP, that is, α1 (Ser5), α4 (Ser130), α5 (Ser198), α6 (Ser110), and β6 (Ser57 and Ser208). O-GlcNAc sites of α1 and α5 are only found in immunoproteasomes, whereas β6 Ser208 modification is detected only in brain proteasomes^66^. O-GlcNAc modification may serve as an important regulation system in the proteasome homeostasis, and investigating the biological impact of O-GlcNAc on proteasome seems to be a challenging but promising field of research.

### Acetylation

Acetylation is a reversible post-translational modification of proteins, regulated by histone acetyltransferases (HATs) and histone deacetylases (HDACs), which add and remove acetyl groups from lysine residues, respectively. Despite the well-documented function of acetylation in modulating gene transcription and protein expression, its role in protein degradation has been recognized. Five lysine acetylation sites on murine 20 S subunits are identified following HDAC inhibition, that is, Lys30 and Lys115 of α6 subunit, Lys77 of β3 subunit, Lys203 of β6 subunit, and Lys201 of β7 subunit (Table 2). The enhanced acetylation of 20 S proteasome subunits leads to an elevation of proteolytic capacity^67^. In this regard, HDAC inhibitors might be a promising class of pharmacological agents for increasing the proteasomal proteolytic activity. Beyond that, six Lys residues (Lys6, Lys11, Lys27, Lys33, Lys48, and Lys63) on ubiquitin have been reported to be acetylated in proteomics datasets^68^. Acetylated ubiquitin does not affect the monoubiquitylation of substrates, but inhibits Lys11, Lys48, and Lys63-linked polyubiquitin chains elongation^69^. Ubiquitin is subject to acetylation to modulate protein degradation, thus providing a new regulatory layer to ubiquitin-proteasome biology. Further research needs to identify the HATs and HDACs responsible for proteasome acetylation and deacetylation, as well as functional roles of acetylated ubiquitin.

## Imbalance of proteostasis in PD

### Proteasome dysfunction exacerbates protein aggregates in PD

**Fig. 3:**
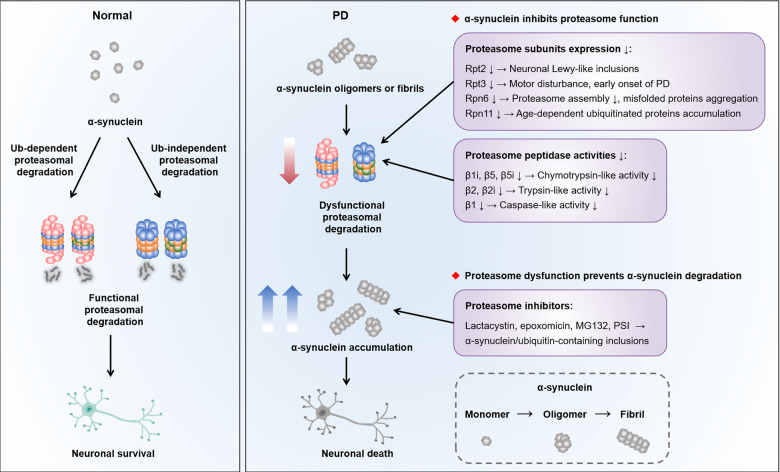
The interplay between proteasome impairment and α-synuclein accumulation in PD. Under physiological state, α-synuclein is highly soluble and enriched at presynaptic terminals, which can be degraded through ubiquitin-dependent and ubiquitin-independent manner, so as to maintain the survival of neurons. In PD, α-synuclein accumulates in neuronal cell bodies to form a major component of aberrant aggregates known as Lewy bodies. α-synuclein is able to transform in different conformations, including monomers, oligomers (soluble conformations), and fibrils (insoluble conformations). The impairment of proteasome function resulting from the decreased subunits expression and proteolytic activities disturbs the degradation of substrates. Additionally, α-synuclein has been reported to inhibit the function of proteasome, which further aggravates the accumulation of α-synuclein. This suggests a vicious cycle between proteasomal impairment and proteotoxicity in PD.

In PD, the selective loss of dopaminergic neurons in the substantia nigra pars compacta (SNpc) and subsequent loss of dopamine in the striatum leads to the classical motor features, such as resting tremor, bradykinesia, rigidity, and postural instability. Although the exact pathogenesis of PD has not been revealed yet, it is generally accepted that disruption of cellular proteostasis is linked to various neurodegenerative diseases including PD^70^. In eukaryotic cells, two major protein clearance pathways, proteasome and autophagy, are interrelated to maintain cellular proteolysis and ensure that cells have the adequate proteins they need^71,72^. The proteasome, in collaboration with a refined ubiquitin system, selectively degrades short-lived proteins as well as misfolded or damaged proteins in the cytoplasm, nucleus and endoplasmic reticulum. By contrast, autophagy coupled with lysosome is essential to degrade longer-lived macromolecules, cytosol fractions, and organelles through three autophagic pathways, macroautophagy, microautophagy, and chaperone-mediated autophagy (CMA). Indeed, maintaining proteome balance is a challenging work in the face of various external and internal stimuli owing to the age-dependent decline in the proteolysis capacity and impaired proteasomal degradation^73^. The resulting aberrant accumulation of misfolded and aggregated proteins probably overload the cellular ability to degrade rendering the neurons susceptible to the pathological injury (Fig. 3).

In 2001, McNaught and colleagues first reported the decreased proteasome hydrolytic activities in PD^74^. Specifically, in the SN instead of other brain regions of PD patients, there is a loss of α-subunits in dopaminergic neurons and a reduction in all of three peptidase activities^75^. In contrast, it is probable that the reduced proteasome activity could be a consequence of the neurodegeneration, which may underly the vulnerability of the SN in PD. ATPase subunit Rpt2 conditional knockout in dopaminergic neurons displays the depletion of 26 S proteasome, resulting in the presence of intraneuronal Lewy-like inclusions in the nigrostriatal pathway^76^. Besides, insertion and deletion variants in the Rpt3 gene correlate with the early onset of PD^77^. Conditional knockout of Rpt3 in motor neurons causes severe motor dysfunction, accompanied by progressive loss of neurons and gliosis^78^. Rpn11 overexpression prevents the age-dependent deficit in 26 S proteasome activity and ubiquitin-conjugated proteins accumulation^79^. Furthermore, Rpn6 enhances the proteasome assembly and activity, which is critical for the stabilization of CP and the proper interaction with RP to improve age-related protein aggregates^26,80^. These data demonstrate that the maintenance of proteasome function is of great importance for the adequate degradation of unwanted proteins and slowing down the neurodegeneration in PD. Even with this information, it is still challenging to pinpoint the different functions produced by the multiple proteasome subunits, which is a major hurdle toward understanding the regulation of proteasome in PD.

### Impairment of α-synuclein degradation through proteasome

Emerging evidence has demonstrated that the abundant fibrillar α-synuclein inclusions known as Lewy bodies is one of the pathological hallmarks in PD. α-Synuclein is encoded by *SNCA* gene, which is enriched in presynaptic terminals to modulate synaptic-vesicle trafficking, bind membranes and induce membrane curvature. Point mutations in *SNCA* (A30P, E46K, H50Q, G51D, A53E, and A53T) and genomic duplications or triplications within *SNCA* locus lead to the autosomal dominant familial PD^81,82^. Indeed, α-synuclein pathology follows a stereotypical “prion-like” propagation pattern, resulting in a cell-to-cell transmission to drive neurodegeneration in PD. Several strategies, such as using antibodies to impede the spreading of α-synuclein and testing minute quantities of α-synuclein in cerebrospinal fluid by seeding aggregation assay, have been implicated for PD therapies^83,84^. The homeostasis of α-synuclein is maintained under intrinsic surveillance mechanisms including ubiquitin-dependent and -independent proteasomal degradation, macroautophagy and CMA. Different forms of α-synuclein are degraded by multiple routes depending on the overall protein burden, localization and pathological states. Proteasome and macroautophagy are capable of degrading mutant α-synuclein or intermediate oligomers, while CMA possesses a specific ability to degrade monomers and dimers of α-synuclein^85,86^. Extracellular α-synuclein is cleared by proteases, or spread among neighboring cells and eliminated within lysosomes. However, the mechanism governing whether α-synuclein will be cleared by proteasome or autophagy remains unknown.

Treatment with proteasome inhibitors leads to the accumulation of ubiquitin and α-synuclein immunoreactive inclusions, which supports the concept that defects in the proteasome pathway might uncover the nigral pathology in familial and sporadic forms of PD^84–86^. Consistent with these findings, systemic exposure to proteasome inhibitors in rats causes the formation of α-synuclein/ubiquitin-containing inclusions resembling Lewy bodies in the remaining neurons and animals develops progressive motor deficits which closely recapitulates the main pathological features of PD^87^. Similar to other PD-related neurotoxins, proteasome inhibitors might offer an alternative way to model the disturbance of protein homeostasis and the chronic progressive nature of neurodegeneration as it occurs in PD. Even so, systemic administration of PSI has been challenged due to several laboratories inability to replicate the mode^88–90^ or reproduce only partial features such as transient motor deficits without dopaminergic neurons degeneration^91^ or modest depletion of striatal dopamine without motor disorder^92^. The impaired turnover of α-synuclein represents a critical aspect of neurodegeneration in PD.

### α-Synuclein disrupts proteasome function

Numerous studies have shown that α-synuclein can inhibit proteasome activity, especially in the presence of oligomer or fibril species^10^. PC12 cells expressing A53T mutant α-synuclein exhibit the impaired proteasomal chymotrypsin-like activity and disruption of the ubiquitin-dependent degradation system, manifested by an increase in ubiquitin-conjugated aggregates^93^. Their observations have been supported by another study using the same cell line expressing mutant α-synuclein, in which the peptidase activities of proteasome are reduced resulting in mitochondrial abnormalities and neuronal death^94^. Of note, proteasome function is inhibited not only by mutant α-synuclein, but also by wild-type species. Human neuroblastoma BE-M17 cells stably expressed wild-type α-synuclein exhibit an ~50% reduction in ubiquitin-independent proteasomal degradation^95^. A study with yeast also demonstrates that wild-type and A30P mutant α-synuclein impair proteasome-mediated protein degradation, but have little effects on intracellular proteasome content or protein ubiquitylation^96^. More recently, McKinnon C et al. report that α-synuclein overexpression leads to the early-onset catalytic impairment of 26 S proteasome, which is associated with selective accumulation of α-synuclein phosphorylated at Ser129 and precedes the onset of motor deficits and dopaminergic neurons degeneration^10^. In addition, α-synuclein oligomers associate with the 26 S proteasome, leading to a significant inhibition of proteasomal activities without affecting the levels or assembly of 26 S proteasome^97^. The proposed mechanisms of how α-synuclein inhibits the proteasome function may be due to the direct interaction with Rpt5 or β5 subunit^95,98^. Overall, the impaired proteasome function aggravates the accumulation of α-synuclein, which in turn binds to the proteasome, thereby inhibiting its proteolytic activity. The vicious cycle between proteasomal impairment and α-synuclein aggregation may provide an insight into the putative neuroprotective therapies for PD.

## Proteasome activation-based approach for PD therapies

### Small-molecule compounds

Enhancing proteasome activity by small-molecule compounds has been regarded as a promising strategy to treat or prevent PD. USP14, a proteasome-associated DUB, contains a catalytic domain at the C terminus and a ubiquitin-like (Ubl) domain at the N-terminus. The binding of USP14 to Rpn1 subunit through Ubl enhances its deubiquitinating activity, which is responsible for the removement of ubiquitin chains and prevent the release of ubiquitin to the proteolytic channel. Proteasomes lacking USP14 exhibit higher peptidase activity and ubiquitin-independent proteolysis^99^. In 2010, Lee et al. have identified IU1 as a selective small-molecule inhibitor of USP14 through high-throughput screen. IU1 treatment significantly accelerates the elimination of oxidized proteins, such as tau and TDP43, thus enhancing the resistance to oxidative stress^100^. Later, the improved USP14 inhibitor IU1-47, with a 10-fold more potent and retains specificity for USP14, has been found to stimulate the degradation of wild-type and pathological tau. Notably, a specific residue in tau (Lys174) is critical for IU1-47-mediated tau degradation^101^ (Table 3). Presumably, further studies are needed to determine the effects of USP14 inhibitors on the proteasomal degradation of α-synuclein in PD.

**Table 3:** Commonly used proteasome activation agents.

Agents	Targets	Initial study results	Clinical and/or other uses	References
IU1/IU1-47	USP14 inhibitor	Proteasome activity ↑; tau and TDP43 degradation ↑	Investigational small-molecule compound	^99,101^
Geldanamycin	HSP90 inhibitor	Oligomeric α-synuclein, phosphorylated tau, and Htt aggregation ↓	Antibiotic; potential use in cancer	^105,106,111^
17-AAG	HSP90 inhibitor	α-synuclein oligomers ↓; Aβ-induced synaptic toxicity and memory impairment ↓	Potential anticancer drug	^108–110^
YM-1	HSP70 activator	Client proteins ubiquitylation and polyQ AR degradation ↑	Investigational small-molecule compound	^112^
DMF	NRF2 activator	Proteasome activity ↑; α-synuclein aggregation ↓	Clinical use in MS treatment	^115,116^
Sulforaphane	NRF2 activator	Proteasome activity ↑; mHtt degradation ↑	Naturally occurring compound	^117^
EGCG	NRF2 activator	α-synuclein fibrillation and aggregation ↓	Naturally occurring compound	^118^
Curcumin	NRF2 activator	Proteasome activity ↑; α-synuclein aggregation ↓	Naturally occurring compound	^119,120^
ASC-JM17	Curcumin analog, NRF1 and NRF2 activator	Proteasome subunits expression↑; antioxidant enzymes ↑	Potential use in SBMA and other polyglutamine diseases	^121^
Lcariin	NRF1 agonist	HRD1 expression ↑; ER stress-induced apoptosis ↓	Potential use in neuronal protection	^122^
sCGA884, sCIN027	NRF1 agonists	Proteasome degradation ↑	Investigational small-molecule compound	^123^
Rolipram	PDE4 inhibitor, cAMP-PKA pathway activation	Proteasome activity ↑; aggregated tau ↓; cognition improvement	Potential use in depression and autoimmune disorders	^124^
PD169316	p38 MAPK inhibitor	Proteasome activity ↑; ubiquitylated protein and α-synuclein degradation ↑	Investigational small-molecule compound	^125^
Nilotinib	Second generation of c-Abl inhibitor	α-synuclein aggregation ↓; normalizing motor disturbance	Clinical use in CML treatment; phase 2 clinical trial of PD	^127,128,132,133^
QC-01-175	Transformation of ^18^F-T807 into tau ligand, coupled with a linker to CRL4^CRBN^	Pathological tau degradation ↑	Investigational PROTAC	^143^

Critical proteins that unfold and aggregate in neurodegenerative diseases, such as α-synuclein, tau, huntingtin (Htt), and polyglutamine androgen receptor (polyQ AR), are client proteins of heat shock protein 90 (HSP90). HSP90/HSP70-based chaperone machinery has been identified as a key regulator of proteostasis owing to its role in the protein quality control^102^. Nonetheless, HSP90 and HSP70 possess opposing effects on client proteins stability. HSP90 prevents the degradation of proteins through the ubiquitin-proteasome pathway, whereas HSP70 promotes ubiquitylation for proteasomal degradation dependent on E3 ubiquitin ligase C terminus of Hsc70-interacting protein (CHIP). This indicates that an effective way to eliminate the aggregation of neurotoxic proteins may be to inhibit HSP90 or promote HSP70 function^103^. Among the compounds that inhibit HSP90, geldanamycin acts as a nucleotide to inhibit the intrinsic ATPase activity of the chaperone^104^. It has been reported that geldanamycin facilitates the degradation of oligomeric α-synuclein, phosphorylated tau, and Htt aggregates in vivo^105–107^. The less toxic analogue of geldanamycin, 17-AAG, has improved brain permeability, which inhibits the formation of α-synuclein oligomer and rescues cytotoxicity mediated by secreted α-synuclein^108^. Furthermore, 17-AAG can reduce tau pathology and attenuate Aβ-induced synaptic toxicity and memory impairment^109–111^. YM-1 allosterically stimulates the activity of HSP70 and enhances its binding to unfolded substrates. Treatment of YM-1 promotes the clearance of polyglutamine proteins and increase CHIP-dependent ubiquitylation of neuronal nitric oxide synthase^112,113^ (Table 3). The compounds that promote HSP70-dependent proteasomal degradation in combination with HSP90 inhibitors might be beneficial for the clearance of aggregated proteins, which should be tested in PD models.

### Transcriptional activation of proteasome expression

Considering that the formation of proteasome is energetically costly, the fine tuning mechanisms are required to maintain sufficient amount of proteasomes to combat the proteotoxicity. Transcription factor NRF2 has been proved to reduce steady-state levels of α-synuclein, shorten its half-life in part by accelerating the degradation of α-synuclein^114^. Pharmacological activation of NRF2 by dimethyl fumarate (DMF), a drug already in use for multiple sclerosis (MS), significantly reduces α-synuclein aggregates and rescues neurons from oxidative stress-induced injury^115,116^. Several naturally occurring NRF2 activators, such as sulforaphane, epigallocatechin-3-gallate (EGCG), and curcumin, stimulate NRF2 signaling pathway to regulate proteostasis. Sulforaphane activates protein degradation machineries and promotes mutant Htt degradation via proteasome^117^. EGCG can inhibit α-synuclein fibrillation and aggregation, as well as protect PC12 cells against α-synuclein-mediated toxicity^118^. Additionally, curcumin has been found to prevent the aggregation of α-synuclein in the dopaminergic neurons^119,120^. A novel curcumin analog, called ASC-JM17, is characterized to strikingly activate NRF1 and NRF2, which further increases the expression of proteasome subunits so as to mitigate the proteotoxicity in spinal and bulbar muscular atrophy (SBMA) and other polyglutamine diseases^121^. Moreover, lcariin functions as an inducer of NRF1 by increasing the expression of HRD1, an ER-anchoring E3 ubiquitin ligase, and protect neurons from ER stress-induced apoptosis in PC12 cells^122^. Recently, high-throughput chemical screen has identified pharmacological agonists of NRF1, that is, sCGA884 and sCIN027. Co-treatment of these activators and MG-132 induce a dramatic transcriptional activity of NRF1. However, there exists an important limitation that single treatment of sCGA884 or sCIN027 leads to only partially efficacious NRF1 transcriptional activation^123^. In this case, a more refined understanding about the upstream signaling governing the transcriptional activity as well as the downstream effects on proteasome subunits expression is critical for targeting proteasome in the treatment of PD.

### Modulation of proteasome phosphorylation

The phosphorylation status of proteasome subunits by PKA, p38 MAPK, and c-Abl has been discussed to modulate the degradation of substrates via proteasome. Administration of rolipram, a specific phosphodiesterase type 4 (PDE4) inhibitor and PKA activator, enhances 26 S proteasome activity, leading to the lower levels of aggregated tau and improved cognitive performance^124^ (Table 3). Later, a proteasome activity-based probe detects 11 novel compounds that enhance proteasome activity, with the p38 MAPK inhibitor PD169316 being one of the most potent molecules. Furthermore, chemical and genetic inhibition of p38 MAPK, its upstream kinases ASK1 and MKK6, and its downstream target MK2, all remarkably increase proteasome activity and the degradation of ubiquitylated proteins as well as α-synuclein^125^. These findings may provide a strategy for investigating the complex biology of p38 MAPK to reduce the aberrant protein aggregates under proteotoxic stress.

The activity of c-Abl, a nonreceptor tyrosine kinase, is increased in the SN of PD postmortem brains and animal models, followed by the accumulation of pathological α-synuclein^126,127^. Several studies have identified α-synuclein and parkin as the substrates of c-Abl, which specifically phosphorylates α-synuclein at Tyr39 and parkin at Tyr143. Currently, the second-generation c-Abl inhibitors Nilotinib and Bafetinib, clinically used in the treatment of chronic myeloid leukemia (CML), have been reported to reduce the accumulation of α-synuclein and reverse the degeneration of nigral dopaminergic neurons in PD animal models^128–131^. Recently, in a phase 2 randomized clinical trial with 75 patients, 150 and 300 mg doses of Nilotinib administered orally once daily for 12 months appear to be reasonably safe, and the dopamine metabolites in cerebrospinal fluid are significantly increased^132,133^. Intriguingly, 150 mg but not 300 mg of Nilotinib has shown a reduction in α-synuclein oligomers^132^ (Table 3). As a consequence, a definitive phase 3 study will be conducted to evaluate the effects of Nilotinib or exploring other c-Abl inhibitors as a potential disease-modifying drug in PD.

### Augmentation of proteasome assembly

The transmembrane domain recognition complex (TRC) pathway, which mediates the insertion of tail-anchored proteins, has been implicated to regulate CP assembly. Bag6, a protein in TRC pathway, facilitates the incorporation of β-subunits into α-ring or directly associates with precursor RP subunits, which is involved in the effective degradation of proteins^134^. In addition, genome-wide functional screening has characterized inactive rhomboid protein 1 (iRhom1), a member of the rhomboid-like family of proteases, as a novel stimulator of proteasome activity^135,136^. Under ER stress, iRhom1 interacts with 20 S CP assembly chaperones PAC1 and PAC2, affecting their protein stability and dimerization. iRhom1 is induced by ER stressors, such as thapsigargin and tunicamycin, and iRhom1 overexpression has been found to significantly enhance the function of proteasome and mitigate the rough-eye phenotype of mutant Huntingtin^136^. However, the precise mechanisms by which TRC pathway controls the proteasome CP assembly or iRhom1 regulates the stability of PAC1/PAC2 dimers remain to be established. NRF3, a close homologue of NRF1, directly augments the expression of proteasome maturation protein (POMP), consequently enhancing the degradation of p53 in a ubiquitin-independent manner^137^. In addition, 19 S base subunit Rpn6 also regulates RP-CP association through the direct binding to α2 subunit. Ectopic expression of Rpn6 is sufficient to enhance proteasome activity and improve resistance to proteotoxic stress^138^.

## PROTAC

PROTAC operates via an event-driven mechanism, which couples a small-molecule binder of a target protein to an E3 ubiquitin ligase via a flexible chemical linker, thereby eliciting ectopic ubiquitylation and eventually leading to the proteasomal degradation^139^. PROTAC is initially implicated in the recruitment of the androgen receptor to the E3 ubiquitin ligase MDM2, and androgen receptor degradation is proteasome dependent, which is mitigated in cells pretreated with proteasome inhibitor^140^. More recently, the field of targeting proteins degradation has expanded dramatically, and PROTACs have been found to be more selective than the intrinsic inhibitors^141,142^. Although p38 MAPK inhibition is implicated to reduce the aberrant protein aggregates under proteotoxic stress, none have displayed the safety and tolerability capable of receiving FDA approval. A recent work has developed p38 MAPK-selective PROTAC based on a single small-molecule binder (foretinib) and E3 ubiquitin ligase von Hippel-Lindau (VHL), which selectively degrades p38α MAPK via proteasome^15^.

Another work designs and synthesizes an effective tau degrader, namely QC-01-175, in which tau positron emission tomography (PET) tracer ^18^F-T807 is transformed into pathogenic tau ligand, coupled with a linker to the CRL4^CRBN^ E3 ubiquitin ligase complex. Intriguingly, QC-01-175 preferentially degrades pathological tau, indicating the degrader specificity for disease-relevant forms of tau^143^ (Table 3). PROTAC may be utilized to develop a functional α-synuclein degrader for the targeted degradation of pathological α-synuclein species in PD where high-quality PET tracers are available. Despite the cytosolic and nuclear proteins can routinely be degraded, the degradation of protein in both the Golgi and ER via PROTAC has not been reported^144^. Indeed, if a PROTAC is designed to induce the degradation of a protein within one system, then it can be applied more widely in different cell types without genetic modification. Regarding clinical applications, off-target effects and appropriate dosage may be an issue, as saturating doses of PROTAC can antagonize the binding of PROTAC-protein complexes to their ternary partner, a well-described phenomenon known as the hook effect in cell assays^145^. The likelihood of successful PROTAC development represents a promising strategy to advance our understanding of neurodegenerative disease and translate those insights into targeted therapies.

## Conclusions

The recent advances in the field of proteasome remarkably improve our understanding of its biological function, well-organized assembly and dynamic regulation of proteasome homeostasis. Under specific conditions, the dissociation and reassembly of subunits to form different types of proteasome, and the distinct function of singly or doubly capped proteasomes need to be further elucidated. In PD, there exists a large number of mutant or misfolded proteins aggregates, which is a testament to the importance of proteasome for proteostasis and its potential as a therapeutic target. Although a variety of proteasome activation strategies have been identified, no drugs that directly enhance proteasome function are available. The elucidation of other compounds to intensify proteasomal degradation will open up new possibilities for PD treatment based on proteasome modulation.
